# Supplementary material for: VEGF Detection via Simplified FLISA Using a 3D Microfluidic Disk Platform
Source: Biosensors (Basel). 2021 Aug 11;11(8):270. doi: 10.3390/bios11080270 (PMC8393963; doi:10.3390/bios11080270)
Supplement: Supplementary file 1 [file biosensors-11-00270-s001.zip › biosensors-1316193-supplementary.pdf]

biosensors

MDPI

Supplementary Materials

VEGF Detection via Simplified FLISA Using a 3D Microfluidic Disk Platform

Dong Hee Kang [1], Na Kyong Kim [1], Sang-Woo Park [2,*] and Hyun Wook Kang [1,*]

[1] Department of Mechanical Engineering, Chonnam National University, 77 Youngbong-ro, Buk-Gu, Gwangju 61186, Korea; kdh05010@gmail.com (D.H.K.); naky0607@gmail.com (N.K.K.)
[2] Department of Ophthalmology, Chonnam National University Medical School and Hospital, Baekseo-ro, Dong-Gu, Gwangju 61469, Korea
* Correspondence: exo70@naver.com (S.-W.P.); kanghw@chonnam.ac.kr (H.W.K.); Tel.: +82-62-530-1662; (H.W.K.) Fax: +82-62-560-1689 (H.W.K.)

Citation: Kang, D.H.; Kim, N.K.; Park, S.-W.; Kang, H.W. VEGF Detection via Simplified FLISA Using a 3D Microfluidic Disk Platform. *Biosensors* **2021**, *11*, x. https://doi.org/10.3390/xxxxx

Received: 2 August 2021
Accepted: 6 August 2021
Published: 11 August 2021

Publisher's Note: MDPI stays neutral with regard to jurisdictional claims in published maps and institutional affiliations.

Copyright: © 2021 by the authors. Submitted for possible open access publication under the terms and conditions of the Creative Commons Attribution (CC BY) license (http://creativecommons.org/licenses/by/4.0/).

Fig. S1 SEM images of microstructure (designed diameter (D) =500, 250, 200, and 100 µm) on 3D printed surface fabricated in the (a–d) vertical and (e–h) lateral directions to show resolution of stereolithography apparatus.

Fig. S2 (a) Top view of 3D-printed block. (b) Isometric view and (c) sectional view of the 3D-printed block.

Fig. S3 Angular velocity of disk with respect to time for the mixing cycle during incubation and the bead washing process.

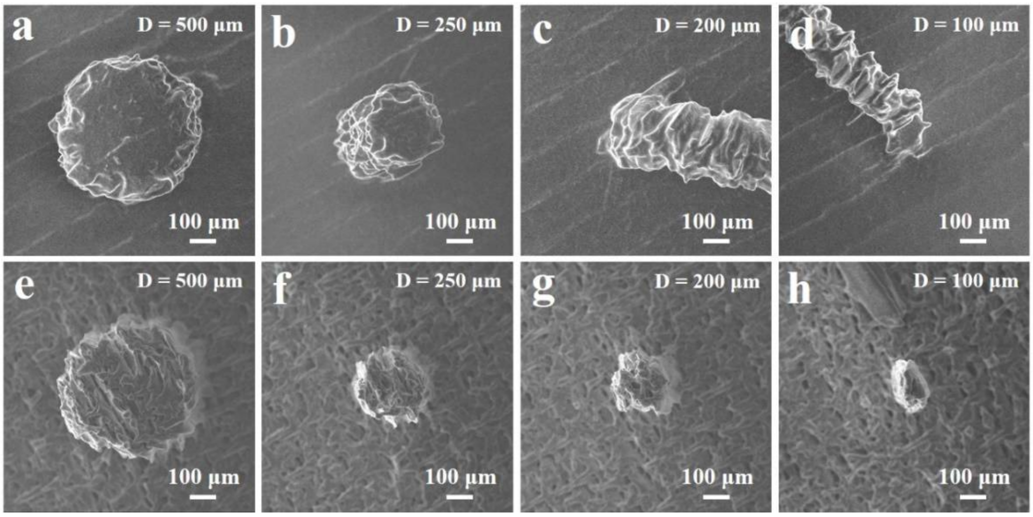

Fig. S1 SEM images of microstructure (designed diameter (D) =500, 250, 200, and 100 µm) on 3D printed surface fabricated in the (a–d) vertical and (e–h) lateral directions to show resolution of stereolithography apparatus.

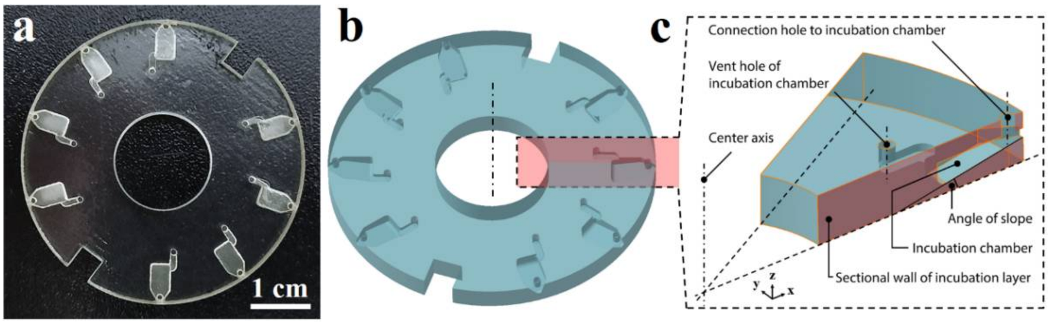

Fig. S2 (a) Top view of 3D-printed block. (b) isometric view and (c) sectional view of the 3D-printed block.

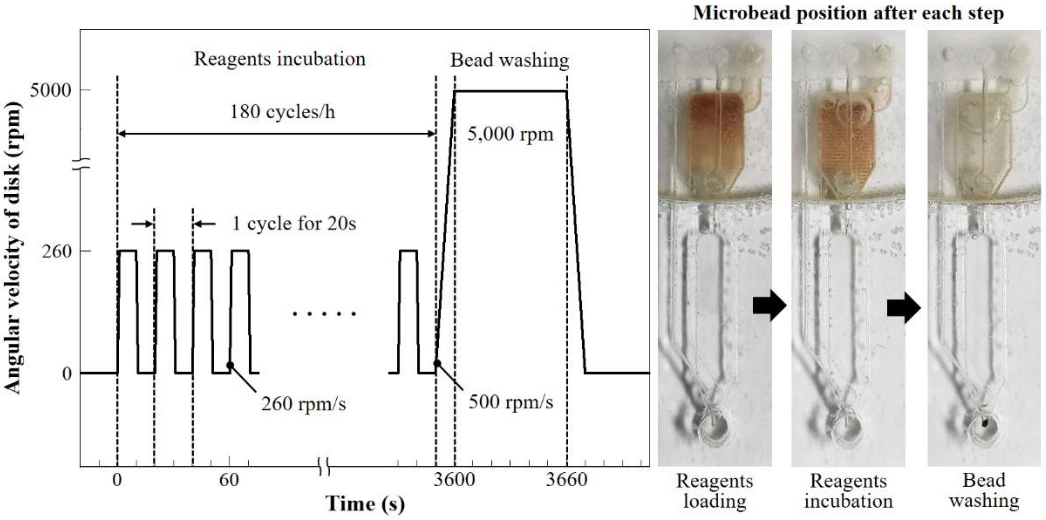

Fig. S3 Angular velocity of disk with respect to time for the mixing cycle during incubation and the bead washing process. The microbead position after each step (reagent loading, reagent incubation, and bead washing).
